# Being the nurse’s eyes and ears: a mixed methods study of assistant nurses’ perceptions of their role regarding drug-related problems in nursing homes

**DOI:** 10.1186/s12912-025-03416-y

**Published:** 2025-06-27

**Authors:** Sarah Thelin, Beata Borgström Bolmsjö, Gabriella Caleres, Astrid Mattsson, Åsa G. Craftman, Patrik Midlöv, Sara Modig

**Affiliations:** 1 Department of Clinical Sciences Malmö, Center for Primary Health Care Research, Lund University, Malmö, Sweden; 2 University Clinic Primary Care, Skåne University Hospital, Region Skåne, Sweden; 3 Department of Medicines Management and Informatics in Skåne County, Malmö, Sweden; 4 Department of Neurobiology, Care Sciences and Society, Division of Nursing, Karolinska Institutet, Stockholm, Sweden; 5 Limhamnsläkarna Primary Health Centre, Apoteksgatan 7, Limhamn, S-216 14 Sweden

**Keywords:** Nursing assistant, Nursing homes, Drug-Related side effects and adverse reactions, Frail elderly, Patient safety, Education, continuing, Mixed methods research

## Abstract

**Background:**

Insufficient knowledge among assistant nurses (ANs) in nursing homes (NHs) on medicines and drug-related problems (DRPs) in older people causes difficulties in acknowledging the side effects of medications, which can be harmful to the individual and endangers patient safety. The aim of this study was to explore the ANs’ thoughts on their professional role in preventing DRPs in NH residents, their self-perceived knowledge about medicines, DRPs and physiological conditions in older people, and their wishes concerning further medical education.

**Methods:**

This mixed methods study took place in Sweden, in 2022. First, a survey was conducted at nine NHs, with a total of 112 respondents. Data were analysed using descriptive statistics and groups were compared using t-tests. Thereafter, semi-structured interviews were performed at four NHs and included 20 participants. A qualitative content analysis was performed.

**Results:**

In the survey, a generally good self-perceived knowledge of medicines was reported. In the interviews, three main categories were identified: “Professional role of the AN”, “Perspectives on pharmacotherapy in older people” and “Approaches to knowledge”. Their professional role entailed different aspects, including the central assignment of observing the residents whilst having delimited responsibilities and knowledge. Regarding pharmacotherapy, the self-perceived knowledge was generally good, but more specific questions regarding DRPs proved a lack of widespread competence. An approach to obtain more knowledge was found in the possibility for ANs to attend ward rounds. A latent theme emerged in the professional role of the AN in preventing DRPs by being the nurse’s eyes and ears for patient safety.

**Conclusions:**

The AN’s perceived their professional role in preventing DRPs in NHs as being the nurse’s eyes and ears for patient safety. Generally, the ANs’ self-perceived knowledge of medicines was good, although self-perceived knowledge regarding DRPs, and the physiology of older people, was sparser. A positive attitude towards attending ward rounds to contribute firsthand information on the residents, as well as the opportunity to receive knowledge, existed among the respondents. We propose a working model within the framework of doctor’s ward rounds at NHs to increase the knowledge of ANs and utilise their knowledge of patients’ well-being for safe medication follow-up.

**Supplementary Information:**

The online version contains supplementary material available at 10.1186/s12912-025-03416-y.

## Background

The population in Sweden as well as internationally is ageing [1–3]. With an ageing population, more people develop diseases and require multiple medications. In Sweden, individuals aged 75 years and older use a mean number of almost six medications, which increases to a mean of 10 medications for individuals living in nursing homes (NHs) [4]. Polypharmacy, i.e. the simultaneous use of several different medications [5], leads to an increased risk of drug-related problems (DRPs) such as side effects and medication interactions [6, 7], along with an increased risk of hospitalisation and death [8]. Changes in the ageing body affect the pharmacokinetics and pharmacodynamics of medications rendering it more difficult to predict the effects and side effects in older people [9] as well as increasing the risk of developing side effects from interactions [10].

Many of the most frail older people in Sweden live in NHs [11], which in 2020 was approximately 108,000 individuals [12]. Frail older people can, due to impaired cognition, dementia or other disease, fail in expressing concerns regarding their health or suspected side effects of medications [13]. Consequently, it is crucial that the personnel working close to the residents in NHs [14] have basic knowledge of medications and DRPs to be able to identify symptoms that could be a sign of medication side effects. In NHs in Sweden, the nurse has the overall responsibility for daily medical care and the unlicensed personnel carry out the main nursing of older people. Typically, a general practitioner from a primary health care centre visits the NH regularly for ward rounds with the nurse. During doctor’s ward rounds, the nurse usually sets the agenda on existing issues to address including reporting suspected DRPs. In addition, a home visit to the resident is carried out to selected residents if required or as a part of periodic follow-up [15].

In this study, the terms assistant nurse (AN) and care assistant (CA) are used to represent unlicensed personnel. The title AN requires an education in health care whereas employment as a CA does not. However, the education for ANs is not standardised, as it can be obtained both at upper secondary level or as an adult education offered by the municipals [16], with equally to one month of full-time studies devoted to anatomy and physiology including pharmacology [17]. Both occupations serve an important function in relieving tasks of the nurse, such as the distribution of medicines, alongside other nursing duties. This practice of executing specific tasks requires delegation. Such delegation is received when the task is ensured to be performed in alignment with the requirements for correct and safe care [18]. Furthermore, an appointed delegation is voluntary for the AN and personal for the individual attaining it, with a one-year limit before renewal is obliged [18–20].

The Swedish National Board of Health and Welfare have defined what is expected of ANs in terms of basic knowledge, namely knowledge of common health problems in older people, knowledge of how ageing affects older people physically and knowledge of medicines and the use of medicines in older people. Furthermore, in-service training for ANs should be offered when needed to maintain their knowledge and abilities [21]. An investigation from the Swedish Ministry of Health and Social Affairs from 2019 observed an extensive lack of competence for ANs regarding, among other areas, medical competence [16]. Presumably, this is due to that employment has been made possible without proper or sufficient training [16]. Inadequate knowledge of medications and DRPs causes difficulties in acknowledging the side effects of medications, which can be harmful to the individual and endangers patient safety [16]. A national report from 2022 found major shortcomings in the distribution of medications at NHs with patient safety risks linked to medication management through delegation [22]. Moreover, Swedish NHs is a field affected by understaffing with high turnover rates and difficulties in recruitment leading to employment of those without proper education and those with limited proficiency in Swedish [12]. Hardly any research is available concerning ANs’ perceptions of medication risks concerning older people and their wishes with regard to further medical training. In light of ANs’ being crucial in the care of older people, including medication management through delegation, and as patient safety risks have been pointed out, more research is needed.

The overarching aim of this study was to explore the ANs’ thoughts on their professional role in preventing DRPs in NH residents and how further medical training can be optimized, with the following research questions:

I) How do ANs perceive their knowledge about medicines, DRPs and physiological conditions in older people?
II) What wishes do ANs have concerning improving their medication-related knowledge?

## Methods

### Study design

The current study used a mixed methods approach. First, a survey was conducted. Thereafter, individual interviews were performed to further qualitatively explore the quantitative findings of the survey.

### Study population

#### Survey

Both ANs and CAs, working at nine out of fifteen chosen NHs were included. All in service were invited to participate and the proportion who declined was negligible. The NHs were selected for geographical distribution from six different municipalities, both rural and urban, in Scania County in southern Sweden. Managers were contacted via e-mail.

#### Interviews

For feasibility reasons, one municipality in Scania County was selected as recruitment base for the interviews. NH managers from the five existing NHs were contacted through e-mail with four accepting to participate. Purposeful sampling of respondents was carried out. One of the NHs had a ward for short-term care. Inclusion criteria were personnel with delegation duties for distributing medications and that had undergone training as an AN with experience of working in NHs regardless of the period of employment. Also, they needed to be available on the suggested dates for the interviews.

### Construction of the survey

The survey was designed by four medical doctors of whom three were PhD-certified general practitioners with a special interest in drug safety. The aim was to explore the self-perceived level of knowledge and need for in-service training of non-licensed personnel at NHs, regarding the physiological conditions of older people in connection with commonly occurring drug treatment and the risk of side effects. It was tested for applicability in a pilot study of 12 respondents from one NH. After the pilot study, minor changes were made to clarify some of the questions. The responses from the pilot study were not included in the main study. In questions regarding the respondents’ self-perceived knowledge, the wording “I feel confident in my abilities regarding…” was used, in opposition to a more direct question: “I have knowledge of…”. These wordings were used as they were deemed to be neutral, non-judgmental and encouraged reflections regarding further medical training. Some of the questions used a 5-point Likert scale where 1 = strongly disagree and 5 = strongly agree, others were multiple-choice or free-text replies.

### Construction of the interview guide

The interview guide was developed by author ST, SM and ÅC with the survey results as a basis and the aim to explore how ANs experience their knowledge of DRPs and thoughts about in-service training. Also, the need to explore how ANs view their own role regarding drug treatment at NHs and safety of care recipients was identified. Furthermore, research on how to best increase the knowledge of ANs about medications and in this way increase drug safety was identified as lacking. The first two interviews were conducted as pilot interviews with readiness to exclude them from the analysis would the need for the interview guide be substantially changed. However, this was not the case.

### Data collection

#### Survey

Collection of the surveys (Appendix 1) took place from February to April 2022. The surveys were paper-based and administered by author AM during visits to the NHs. Ten of the visits were done in-person, whilst one for practical reasons was given digitally with information through Microsoft Teams with the filled in paper-based surveys later posted. A presentation of the survey and its purpose was made on each occasion and the possibility to ask questions whilst answering the survey was given.

#### Interviews

The managers of each NH were asked to identify personnel that met the inclusion criteria. Prior to the interviews, the personnel who accepted the invitation received an information letter describing the study and the purpose of the interviews, as well as the voluntary nature of participating. Contact details for the researcher conducting the interviews were provided and written consent was collected. Semi-structured interviews were conducted, using an interview guide (Appendix 2) by author ST at the NHs, and took place between September and November 2022. The interviews took place during the respondents working hours in an undisturbed location at the NHs and lasted between 20 and 50 min each. The interviews were audio recorded and transcribed verbatim.

### Analysis

#### Survey

The survey data were analysed using descriptive statistics. Questions following the Likert scale are mainly presented with median numbers given primarily being based on an ordinal scale. However, it is argued that the Likert-scale can be interpreted as an interval-scale with the same distance between the different answering alternatives [23]. Thus, the questions are also presented with mean numbers. The means of different groups of responders were compared using t-tests. Grouping was based on the amount of time in the profession and the type of section in the NHs the respondents worked at. Statistical significance was set to *p* < 0.05. For the survey’s free text questions, summative content analysis was applied. The response rate of each question was noted and missing answers and answers with ‘Not relevant/Do not wish to respond’ were not included in the summation of number (n).

#### Interviews

A qualitative content analysis was performed, inspired by Graneheim and Lundman [24]. Initially, the first 15 interviews were checked for correctness against the corresponding audio file. Thereafter the analysis began with the first author reading the interview transcript several times through to obtain an overall sense of the content of each individual interview. Next, meaning units reflecting the aim were distinguished, condensed, and subsequently coded based on their content. SM, GC, BB, ÅC and PM each read one interview as well as the coding for confirmation. The first step in categorizing the codes was a discussion amongst the authors based on the initial impressions of the interviews. Thereafter, subcategories were developed based on the substance of the codes and finally, the main categories were revised and established. Table 1 shows an example of the analysis process.

**Table 1:** Example of the analysis process

Meaning unit	Code	Subcategory	Main category
”…I react strongly to it, here is something that is not right…either the parameters or you check whether there are any new medications or something, you discuss with the colleagues…then you can call the nurse…and then she can check it out in a different way, so we know how to solve the problem.” (Participant 13)	React to a change in the resident’s mood by contacting the nurse	Observing residents	Professional role of the assistant nurse
“…if you consider quality of life, it doesn’t matter, it sounds harsh, but if you can be helped by Paracetamol, even though you risk liver damage etc…so you have to weigh pros and cons there.” (Participant 17)	It may be worth risking serious side effects if it increases quality of life	Conceptions about polypharmacy	Perspective on pharmacotherapy in older people
“…you get an insight into how the doctor thinks, they say things about things that might not come up otherwise and you get an insight into why you remove or add a medicine and how she thinks.” (Participant 2)	Cooperation in ward rounds contributes to increased knowledge about medicines	Collaboration as a source of knowledge and information transfer	Approaches to knowledge

After the initial analysis, the remaining five interviews were coded in the same manner as described above. The other authors read several additional interviews, thus confirming the accuracy of the analysis as well as ensuring the saturation of the material. Finally, the manifest content was confirmed, and a latent theme was distinguished.

### Ethics

Participants were given written and verbal study information, including the voluntary nature of participation and the right to withdraw, as well as the perseverance of their confidentiality and the anonymisation of their quotes to protect the identity of the participants. Informed consent was obtained from all participants. Accordingly, these conditions follow the recommendations of the Swedish Research Council [25]. The Swedish Ethics Review Authority assessed that no ethical approval was needed, instead an advisory remark was obtained (Registry number 2021-06597-01) stating that there were no ethical objections to the study. The research followed the Declaration of Helsinki ethical principles [26]. Furthermore, the researcher conducting the interviews (ST) was not involved in the medical care at the NHs.

## Results

### Survey

A total of 112 questionnaires were answered on 11 different survey occasions. The dropout rate consisted of one questionnaire that was not delivered by post from the NH where the survey collection visit took place virtually. There was no substantial missing data. The background data for the respondents is described in Table 2.

**Table 2:** Background data for survey participants

Background data, *N* = 112	*n* (%)
*Legal sex*,* n = 112*	
Female	103 (92)
*Age*,* n = 111*	
25 or younger	8 (7.2)
25–34	22 (19.8)
35–49	32 (28.8)
50 or older	49 (44.1)
*Language*,* n = 111*	
Speaks and understands Swedish fluently	109 (98.2)
*Has education as an assistant nurse*,* n = 112*	
Yes	91 (81.3)
*Occupational experience*,* n = 112*	
1 year or less	1 (0.9)
1–5 years	26 (23.2)
6–15 years	38 (33.9)
15 years or more	47 (42.0)
*Holds permanent employment*,* n = 112*	
Yes	98 (87.5)
*Type of department*,* n = 111*	
Dementia department	27 (24.3)
Regular department	79 (71.2)
Both dementia and regular department	5 (4.5)
*Has additional training with older people*,* n = 111*	
Yes	50 (45.0)
*Offered introductory training at workplace*,* n = 109*	
Yes	78 (71.6)
*Has delegation to distribute medicines*,* n = 112*	
Yes	108 (96.4)
*Has received training before obtaining delegation for distributing medications*,* n = 112*	
Yes	101 (90.2)
No	9 (8)
Does not have delegation	2 (1.8)

### Knowledge

The respondents indicated that it is important to know which medicines their resident takes and why (Table 3). No significant differences were seen between the group who had worked for a long (*≥* 6 years) versus a shorter time in the profession regarding self-perceived knowledge of the medicines that the residents take (question 1) and self-perceived knowledge of potentially inappropriate medicines (PIMs) for older people (question 5). Nor did the type of department the respondent worked on (dementia/regular) make any significant difference regarding these questions.

**Table 3:** Respondents’ self-perceived knowledge

Questions*	Mean value	Median
1. I feel confident with my knowledge of the medications that my residents take. (*n* = 107)	4.0	4
2. I feel confident with my knowledge of side effects that may occur because of medications. (*n* = 106)	3.6	4
3. I often feel worried that problems will arise when new medicines are introduced. (*n* = 106)	2.3	2
4. I believe it is important to know which medicines my resident is taking and why. (*n* = 108)	4.5	5
5. I feel confident with my knowledge of which medication groups are classified as potentially inappropriate for older people. (*n* = 105)	3.4	3
6. I feel confident with my knowledge of which medication groups pose a risk of causing falls. (*n* = 107)	3.8	4
7. I feel confident with my knowledge of which medicines that can pose a risk and need to be paused (after doctor’s consultation) in case of stomach upset or high fever, i.e., in case of risk of dehydration/acute kidney failure. (*n* = 105)	3.3	3
8. I feel confident with my knowledge of the pros and cons of sleeping medication. (*n* = 106)	3.9	4
9. I feel confident with my knowledge of other methods (apart from medications) that can be used to improve sleep in older people. (*n* = 105)	4.1	4
10. I feel confident with my knowledge of other methods (apart from medication) that can be used to reduce anxiety in older people. (*n* = 108)	4.0	4
11. I have knowledge of how malnutrition can be detected and prevented in older people. (*n* = 110)	4.2	4
12. I believe that medication is the most important treatment available for symptoms that can occur with ageing. (*n* = 100)	2.7	3

*Likert scale score 1–5, 1 = Strongly disagree, 5 = Strongly agree

**Fig. 1:**
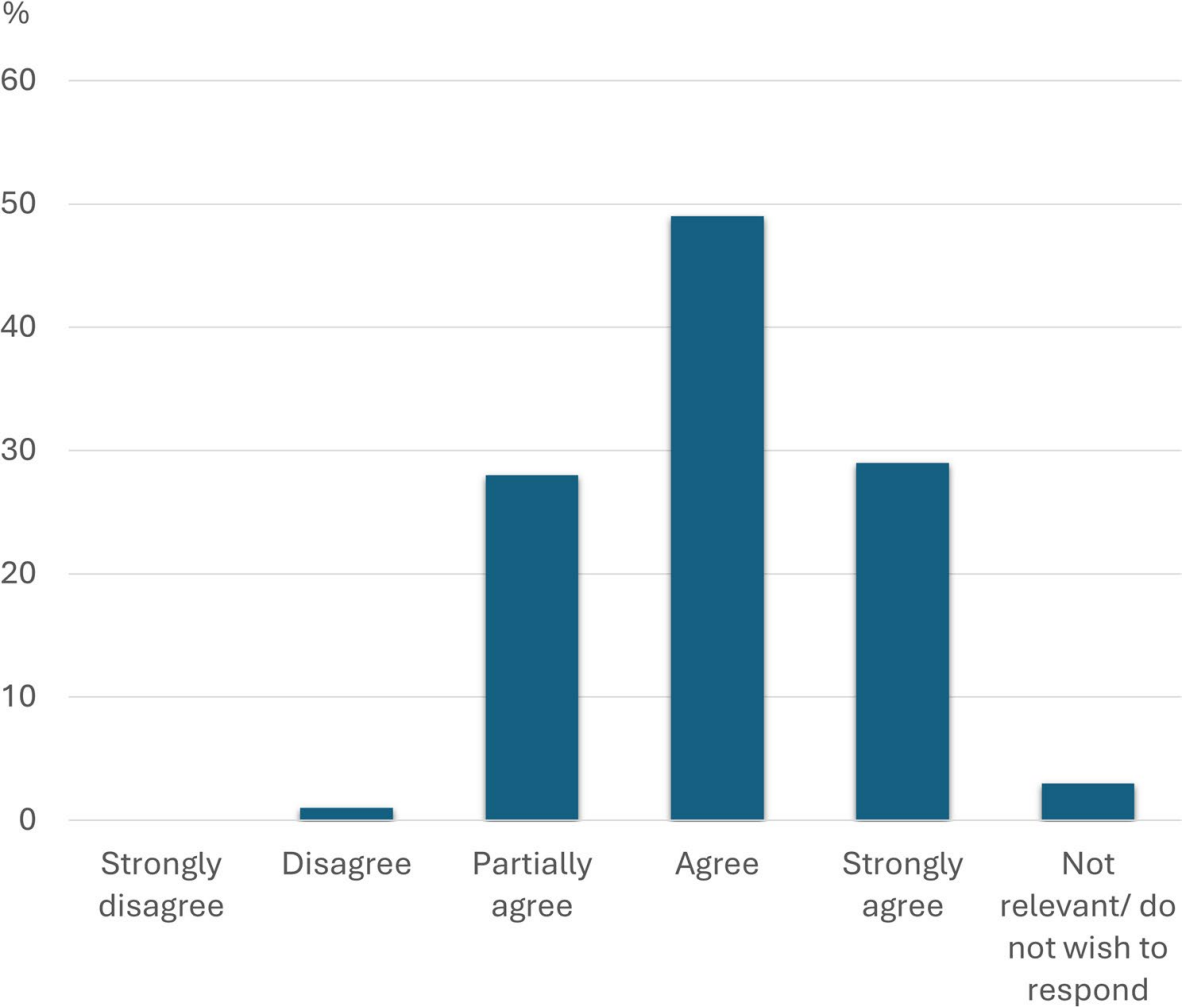
Knowledge of medications. Distribution of how the participants answered question 1 “I feel confident with my knowledge of the medications my residents take” (*N* = 107)

**Fig. 2:**
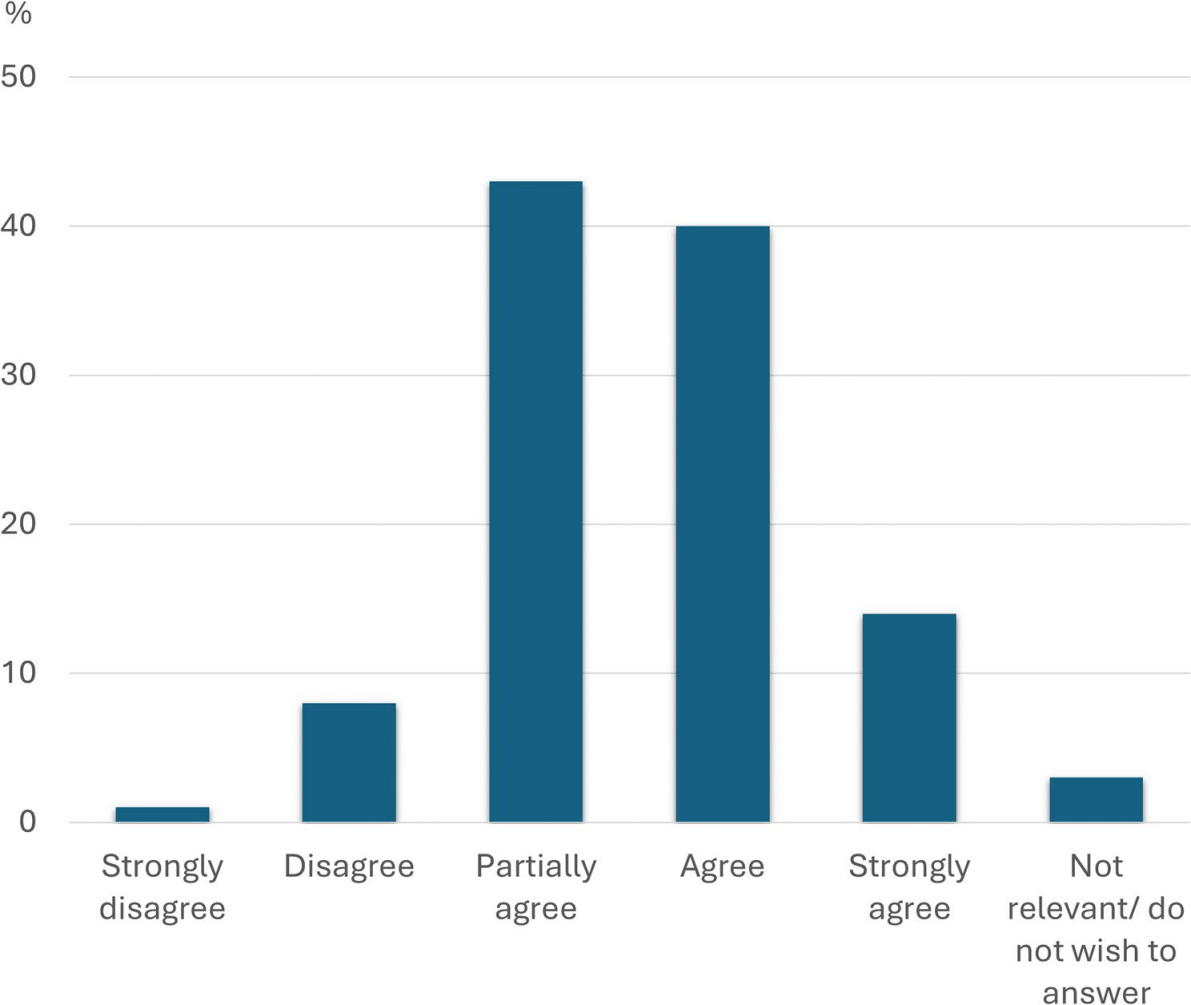
Knowledge of side effects. Distribution of how the participants answered question 2 “I feel confident with my knowledge of side effects that may occur because of medications” (*N* = 106)

**Fig. 3:**
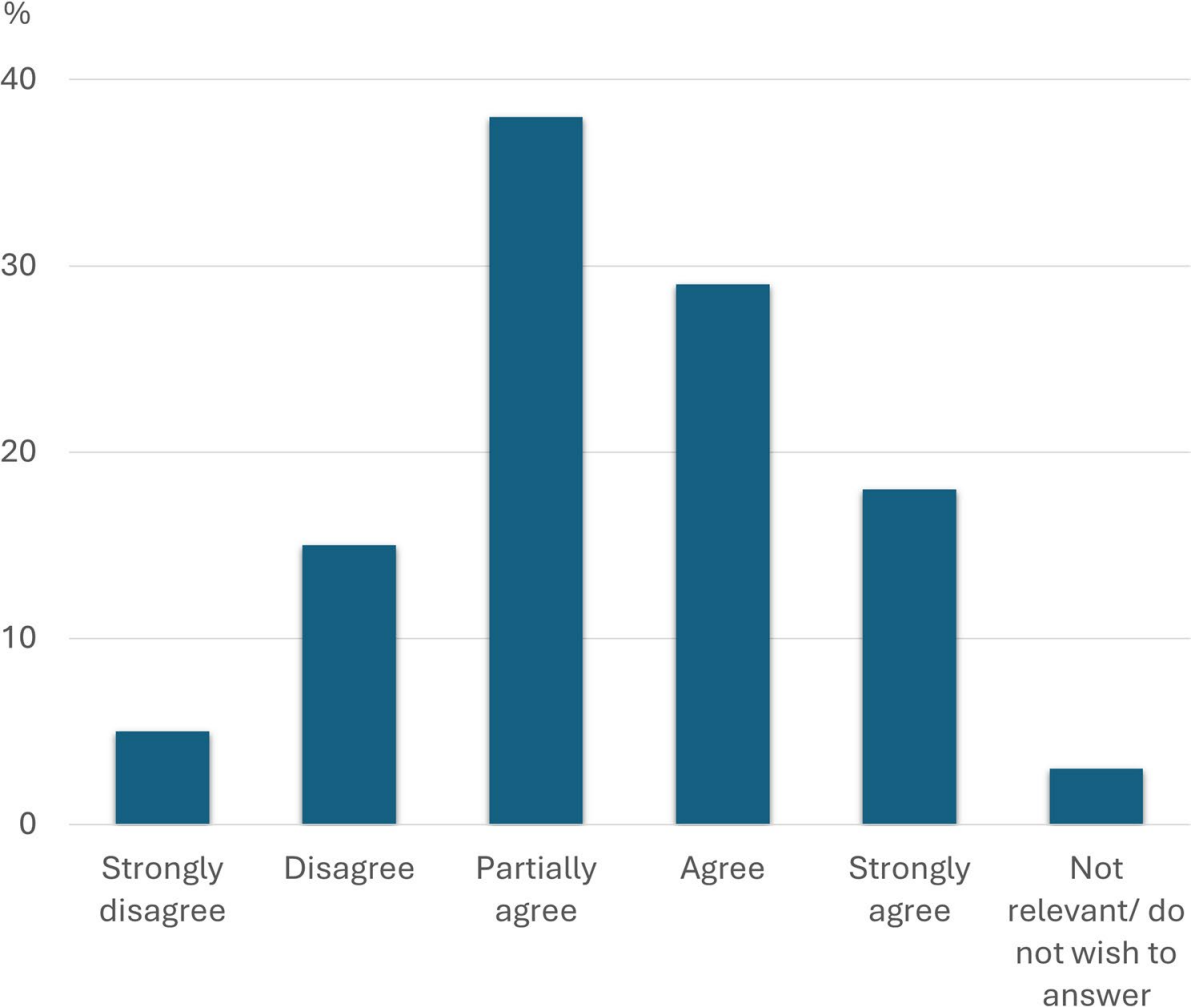
Knowledge of potentially inappropriate medications for older people. Distribution of how the participants answered question 5 “I feel confident with my knowledge of which medication groups are classified as potentially inappropriate for older people” (*N* = 105)

Figures 1, 2 and 3 below show in detail how the respondents answered three of the key questions from Table 3 regarding self-perceived knowledge of medications, side effects and PIMs. Also, a total of 75% of the respondents answered that they had at some time suspected a medication side effect in a resident, and 83% stated that they had reported further suspicions of the side effect to, for example, the nurse.

**Fig. 4:**
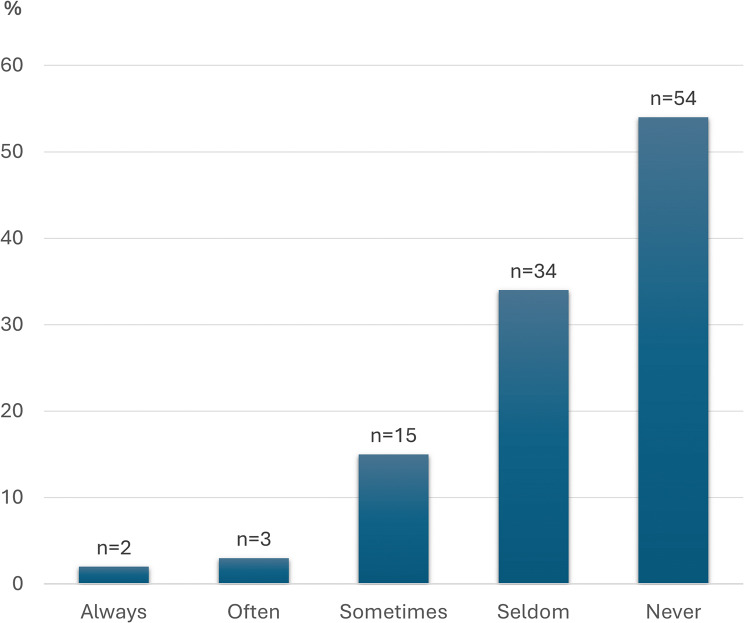
Do you participate in ward rounds visits with doctors? Distribution of how often the respondents participate in ward rounds visits with doctors (*N* = 108)

Figure 4 shows how often the respondents participated in ward rounds visits with doctors. 81% of the respondents reported to rarely or never participating in ward rounds visits. The reasons given for not participating were not being invited (81%), lack of time (7%), having nothing to contribute (3%), and other reasons (9%). Similarly, 79% of the respondents reported to rarely or never participating in multiprofessional medication reviews performed by a multiprofessional team consisting of clinical pharmacist, GP, and nurse at the nursing home [27]. Participation in ward rounds visits and in multiprofessional medication reviews was considered important by most of the respondents. Both being able to share their experiences of the resident’s well-being and be informed of the doctor’s/pharmacist’s rationale for the resident’s medication treatment was considered important, as well as learning by their participation. Between 60 and 69% of the respondents agreed with statements concerning the benefits of participating in ward rounds visits and medication reviews.

### In-service training

A total of 60% of the participants had taken part in in-service training in the past year. Reasons for not receiving in-service training in the last year were reported to be due to not being offered in-service training (60%), not being allowed to go during working hours (6%), already having knowledge of the in-service training theme (4%), lack of time (2%) and other reasons (16%) where several participants mentioned the Covid pandemic as the reason for not having attended any continuing training in the past year. Furthermore, 49% of the participants had at some point been offered in-service training on medication management. Regarding in-service training on pharmacotherapy, 19% had been offered in-service training about medication side effects and 15% had been offered in-service training about age-related changes in the organs that affect medication sensitivity. In addition, 63% had been offered other in-service training related to ageing, such as dealing with dementia. Most of the respondents had received their in-service training as online education, with the rest as in-person or digital lectures.

There were 72% of the participants who indicated that they wished for more in-service training in the future. The preferred means of accessing in-service training was through in-person courses (84%), digital courses (31%), daily discussions with authorised personnel (28%), following a nurse (25%) and other (5%). In the free text comments about additional thoughts regarding in-service training, there were suggestions such as *“more focus on side effects/clashes between medications”*, *“other methods to reduce worry*,* malnutrition*,* anxiety and sleep problems”* and *“more training on dementia (lectures)”*. The collaboration with nurses and doctors was also commented on. One participant wrote that a fantastic collaboration with the nurse was their safety, while another mentioned that a lot (of information) goes through the nurse and that the information sometimes does not reach the AN. Another wished to be more involved in the collaboration with nurses and doctors. Two seemingly experienced respondents commented: *“Different levels among the staff. Give continuing training to those of us who have a high level of knowledge*,* not always basic level.”* and *“It has become more common with education in recent times. When I started in healthcare*,* it was “easier” to get delegation. There were no educations then.”*.

### Interviews

In total, twenty participants were interviewed. The respondents were between 27 and 64 years old, 17 were female and three were male. Two had additional training in palliative care and seven were employed as team leaders. The time spent in the profession ranged from two to 38 years, and the number of years at the current workplace was between less than one year to 37 years.

Three main categories were identified when analysing the interviews: “Professional role of the AN”, “Perspectives on pharmacotherapy in older people” and “Approaches to knowledge”. The first category focuses on the different roles associated with being an AN. The perspectives on pharmacotherapy were based on conceptions about potentially harmful medications, polypharmacy, and side effects, along with perceptions of medication prescription and follow-up as well as conceptions about the physiological conditions of older people. Approaches to knowledge implied self-perceived knowledge, perceptions on acquiring knowledge and collaboration as a source of knowledge and information transfer. (Table 4)

**Table 4:** Main categories and subcategories

Main categories	Subcategories
Professional role of the assistant nurse	Contribution to safe medication management
	The process of delegation
	The observer
	Identification and action on potential side effects
	Delimited responsibility
	Professional integrity
	Nurse as a manager and supporter
Perspective on pharmacotherapy in older people	Conceptions about potentially harmful medications
	Conceptions about side effects
	Conceptions about polypharmacy
	Perceptions of medication prescription and follow-up
	Conceptions about the physiological conditions of the older adult
Approaches to knowledge	Reflections on self-perceived knowledge
	Perceptions on acquiring increased knowledge
	Collaboration as a source of knowledge and information transfer

### Professional role of the AN

#### Contribution to safe medication management

The respondents stressed the importance of the medication list being accurate. Likewise, they emphasised exerting precision in adhering to the medication list.

*[The most important thing is] that the medication list is correct. (…) You can’t give something that isn’t right*,* or you’re not allowed to give something that isn’t right*,* that’s just the way it is.* (Participant 7)

If the medication list was not correct, the nurse was contacted. Furthermore, the ANs’ wanted to know why the residents took their medicines. Information could be difficult to obtain if it was not stated in the medication list.

Careful attention was given to the correct distribution of medicines. Additionally, sometimes extra attention regarding medicines was needed, for instance when distributing anticoagulation medication or due to temporary changes. Digital aids were mentioned as tools for facilitating medication management safety. However, sometimes it was not practically possible to follow all recommended routines, such as giving the medication in the resident’s room.

Several risks in the management of residents’ medication were described, such as the risk of errors when operating with multiple medication lists, being interrupted in their work, risks due to colleagues not working uniformly and insufficient time. Also, the residents themselves may deviate from the medication list by saving pills instead of taking them when administered. Actions for dealing with mistakes in medication management were taken and included documentation and contacting the nurse.

#### The process of delegation

The process of delegation involved an annual knowledge test, which could cause discomfort and stir up different emotions. Some regarded the test as boring and unnecessary, whilst others described a feeling of not being respected for their knowledge.

*And compared to the substitutes who come*,* you take the same test as the substitutes who have never heard anything about medicine. I can be. you can be simply sad and disappointed*,* that you matter the same as the person who knew nothing about medicine.* (Participant 3)

However, some found it beneficial. There was a wide range in how the test was perceived, from difficult to basic. Furthermore, several respondents wanted additional delegation tasks, such as taking blood samples or the placement of urinary catheters. Accepting delegation was voluntary and provided the possibility of an opt-out. However, having delegation was considered as an act of solidarity.

#### The observer

The observing role could be both on the ANs’ own initiative and an assigned task. The respondents stressed the importance of reacting to a change in the resident’s mood and reporting it to the nurse.

*If something changes with a resident*,* then they usually say that we know the resident*,* yes*,* they don’t look the same as yesterday*,* for example. (…) It is something*,* check vital parameters. (…) Blood pressure*,* temperature*,* and all that. Then we give it to the nurse*,* then what they do*,* sometimes they can say yes*,* we will continue observing.* (Participant 1)

However, the cause of the sudden change in the resident’s general condition did not seem to be of deeper interest to the ANs’. There were situations when extra observation was needed, for instance in the case of newly introduced or changes in the medicine as well as when distributing medication with an increased risk for older people. A time delay could occur when it was not the same staff that was observing continuously.

#### Identification and action on potential side effects

The respondents reported that they primarily contacted the nurse when they observed a suspected medication side effect.

*We’ve had one who was given some painkillers that made him very sick*,* he felt nauseous. And that’s the kind of thing we see when a new pill is introduced. Then we notice it and talk to the nurse about it. Some [medicines] have side effects*,* they can cause nausea and so on.* (Participant 15)

A few examples of reading up on, talking with colleagues or contacting relatives also occurred. In addition, an example of taking independent actions in an emergency situation with hypoglycaemia existed.

#### Delimited responsibility

The role of the AN was clearly regarded as delimited when it came to their responsibilities, where the nurse had the main responsibility for assessments and medications on the basis that she had more knowledge. The ANs did not consider themselves responsible for knowledge of medicines, their side effects or interactions and clearly abstained from responsibility. However, they felt a responsibility to react to changes in the mood of residents and took responsibility for their care and daily routines.

*What I can contribute with are my eyes and vision*,* and hearing*,* and inform and reconnect if there are any changes [in the resident].* (Participant 13)

Such responsibility lay especially with permanent staff, and team leaders had additional responsibilities. Furthermore, they could inform the nurse if they believed medications should be reviewed or if they did not think the medical care was good enough. Such reporting was necessary to stay clear of responsibility for irregularities.

#### Professional integrity

The ANs emphasised the meaningfulness of their work where the residents became like family members. They described the importance to acknowledge the person and be there to help them and make sure they got a noble final stage in life. Furthermore, in ward rounds, the ANs could provide support and security for the residents and were able to help them describe their problems.

Also, they mentioned the importance of having the resident’s trust and their role in motivating the resident to take their medication. However, when there was a mistake in medication management, their overall job satisfaction decreased. They described appreciation and felt respected for being involved in the professional collaboration in ward rounds and the importance of the recognition of the ANs’ role, as exemplified here:

*Yes*,* you contribute a lot*,* (…)…when some drug was lowered because I said that there had been improvement*,* and yes*,* I felt I contributed.* (Participant 20)

#### Nurse as a manager and supporter

The nurse was perceived as the spider in the web regarding medication issues. The ANs expected the nurse to answer questions, inform about possible side effects of medication and risk situations related to medication distribution, as well as to be the link between the ANs and the doctor and explain incomprehensible facts when different professions communicate with different vocabulary. One respondent commented:

*If a patient has dementia*,* the nurse tells us*,* for example*,* if Memantine is introduced*,* she says that for the first two weeks she will feel a bit bad and a bit more anxious and stuff like that*,* so she prepares us.* (Participant 4)

Communication with the nurse was considered important and substitute nurses were perceived as a potential patient risk. All respondents stated that they contacted the nurse with questions regarding medicines.

### Perspective on pharmacotherapy in older people

#### Conceptions about potentially harmful medications

Awareness of several different groups of potentially harmful medications and to some extent which organs are affected existed. However, there were also notions that all medicines were potentially harmful medicines and that harmless medicines were potentially harmful medicines, for instance, paracetamol was often mentioned in this context.

*They have their two [Paracetamol] at 8*,* 14 and 20. so it’s the same and you are not supposed to have it for more than 14 days.* (Participant 13)

Uncertainty emerged about what was meant by a potentially harmful medication and there was a lack of ability to identify a potentially harmful medication.

#### Conceptions about side effects

Several respondents gave relevant examples of side effects of various medicines, but a majority could not upon questioning give an example of an observed medication side effect. Uncertainty about what constitutes a side effect occurred, including examples of when the side effect was only equalised with allergy and when the indication for the medicine was perceived as a side effect.

*…we don’t give medicines just like that*,* the doctor goes through everything*,* then the nurse*,* then the nurse asks us if they have an allergy*,* do they have that*,* [an allergy] that is*,* and then the nurse usually says that; you have to be careful. so very careful there*,* you have to keep an eye if it affects [the resident]*,* [then] you should contact me. That’s the reason*,* but I’ve never seen any kind of reaction*,* no.* (Participant 6)

One respondent pointed out that it may be worth risking serious side effects if it provides increased quality of life.

#### Conceptions about polypharmacy

The notion that residents have many pills emerged. Some were perceived as unnecessary and the need for them was questioned. Also, the increased risk for side effects and medication interactions posed a concern.

*…some of them take so many pills*,* you kind of wonder how they find the right one in the system and if you should review them*,* if one outweighs the other and the side effects it gives*,* you would think.* (Participant 20)

However, it was admitted that sometimes many medicines were required, and that residents must receive the medicines they need.

#### Perceptions of medication prescription and follow-up

The respondents described the doctor as being responsible for prescribing medicine and stressed the importance of the prescription being based on correct assessment. However, critical opinions emerged that follow-up and discontinuation of unnecessary medicines were made too rarely and that it took too long before doctors acted on problems that had been remarked upon by the AN. Furthermore, it was also perceived that natural supplements were not always noticed, and that the resident had poor compliance if there was a lack of trust in the doctor. On the other hand, the assessment of medications was described as having improved compared to earlier praxis and that withdrawal of medicines was tried if side effects were suspected.

*If I compare with before*,* in the past that is*,* I think the follow-up is much better*,* much better. In the past*,* they started a medication and then that was it. Now it is removed*,* and you evaluate*,* and you do annual checks and so on and it was not done in the same way before*,* so I think it has become much*,* much better.* (Participant 2)

#### Conceptions about the physiological conditions of older people

Knowledge of physiological changes in several different organ systems in older people emerged, ranging from general medication sensitivity to specific knowledge of changes in organ systems.

*…they often react more to medicines than we do*,* because they don’t have that. that cleansing process in the body either anymore*,* because the body declines when you get older*,* that’s just the way it is.* (Participant 7)

However, several respondents could not suggest any physiological change at all in older people. Also, some examples were incorrect or incomplete and of less relevance while others were adequate examples of altered sensitivity in certain organ systems.

### Approaches to knowledge

#### Reflections on self-perceived knowledge

Most of the respondents reported having taken various in-service training courses in a variety of subjects related to the role of the AN. Several stated that they ensconced knowledge and had not received it through in-service training. A general good self-perceived knowledge about medicines and older people was reported, which contributed to reduced uncertainty and the sense of being competent.

*And then the relatives also have some questions and of course*,* if we can’t answer them*,* we’ll pass it on to the nurse. But it still feels good to be able to answer. That they feel that yes*,* but we are competent*,* we know this.* (Participant 2)

Several respondents pointed out that the ANs’ knowledge is limited and that they have practical knowledge which resides in the management of medications rather than the different types of medicines. However, among other respondents were feelings of a lack of knowledge and education in both themselves and in colleagues.

#### Perceptions on acquiring increased knowledge

The respondents expressed that they could acquire knowledge from primarily the nurse, but also from colleagues, the patient leaflet, and the internet, such as the online database for licensed medicines. The notion that it is important to learn from your mistakes and to be curious and ask questions emerged.

*And then I’m very curious and inquisitive*,* so what I don’t know*,* I find out and ask.* (Participant 17)

However, some stated that they refrain from obtaining information and that it sometimes was difficult to find information. Furthermore, experiencing a lack of time to obtain information was also described.

Concerning in-service training, a lack of time, practical conditions, and user dependence such as the presence of a language barrier, digital unfamiliarity, and inability to concentrate, appeared as obstacles. Furthermore, uncertainty about what education was offered and how you qualify to take part in it was mentioned. Education was often initiated by the manager as part of mandatory duties. The respondents were generally positive towards the in-service training they have taken part in and found it rewarding to learn new things. However, some did not find the in-service training stimulating enough, and some did not wish for more in-service training. There were requests for in-service training in a variety of areas in different forms; as a digital or in-person course, or as practice or walking side-by-side with a nurse.

#### Collaboration as a source of knowledge and information transfer

Often, no feedback was given on what the resident’s change in mood was due to. ANs were neither obvious participants nor expected to participate in ward rounds work. However, the general attitude towards participating was positive. The collaboration during ward rounds work contributed to new knowledge and gave the ANs the opportunity to ask questions. Furthermore, it also meant the opportunity to contribute with decision-making information in medical matters, as the ANs have up-to-date information and advantageous personal knowledge of the residents.

*[To contribute during ward rounds] one hopes that one could have done perhaps. It is after all us who know our patients the most*,* than nurses and doctors do*,* they don’t*,* they only see [medicines]. we see everything; we’re with them all the time*,* all the time.* (Participant 19)

Different experiences from participating in ward rounds occurred; both the impression of being heard, but also the notion of not being heard for one’s reporting. Closer cooperation with the doctor was requested and the awareness that different professions have different inputs, as well as the importance of teamwork.

### Latent theme in the results– being the nurse’s eyes and ears for patient safety

A latent theme was identified with the AN as the observer - the eyes and ears of the nurse - with the aim of maintaining patient safety. We found that the ANs were both aware and proud of their important role as the nurse’s observer. They were confident in their ability to observe correctly and there was an awareness that they are essential to do so due to their close relationship with the residents. Simultaneously, they maintained their limited assignment which did not involve interpretation of the observations, instead the nurse and doctor were appointed with that responsibility. A great respect for the well-being of older people and the ANs’ participation in maintaining their safety became apparent.

## Discussion

### Main findings

This was, to our knowledge, the first study on the self-perceived knowledge of medications and DRPs as well as thoughts about in-service training among ANs at NHs. In the survey, a generally good self-perceived knowledge of medicines was reported. Most of the respondents were positive towards attending ward rounds even though a vast majority seldom or never did. Three main categories reflected the results of the interviews: “Professional role of the AN”, “Perspectives on pharmacotherapy in older people” and “Approaches to knowledge”. Different aspects of working as an AN appeared including the central assignment of observing the residents whilst having delimited responsibilities and knowledge. Corresponding to the survey, the self-perceived knowledge of medicines was generally good but more specific questions regarding DRPs proved a lack of widespread competence. Also, a positive attitude towards attending ward rounds to contribute with first-hand information on the residents, as well as the opportunity to receive knowledge, existed among the respondents.

A latent theme emerged in the professional role of the AN in preventing DRPs by being the nurse’s eyes and ears for patient safety. Their close relationship with the residents allows them to be able to observe them adequately. Their observations are vital for improving patient safety with regard to how medications impact the residents. However, to sufficiently make use of the knowledge of the ANs’, it should be conveyed further to the nurse and the doctor, possibly by participating in the doctor’s ward rounds, where the AN could both provide the doctor with first-hand information on the residents and could also be informed of potential DRPs they should be observant of.

### Professional role of the AN

Both in the survey and interviews, the practice of having delegation for distributing medication was an integrated aspect of being an AN. A Swedish study on nurses’ experience regarding the delegation of medication distribution to ANs and CAs described that the nurses often feel pressured to give delegation even when they are unsure whether it is safe to delegate the task to the person in question [19]. In a study on home care, the ANs and CAs described that they perceived medication distribution as an integrated part of the nursing work - even when they were unfamiliar with the administered medications. On certain occasions, the respondents also felt not being taken seriously when, for example, they reported a change in the health status of a resident to another care profession [28]. This supports the findings in our study where it seems to be anticipated that ANs have delegation to distribute medicines but are not expected to participate in ward rounds with the doctor to share their thoughts and observations about the health status of the residents.

The AN, nurse and doctor have different backgrounds and “speak different languages”. This could be a reason that the implication of follow-up, in-service training and side effects were not obvious to the interview respondents. A review on interprofessional communication in hospitals found that developing strong working relationships with colleagues from other professions was considered an important facilitator for communication between different professions [29]. Furthermore, the importance of adequate communication between health care professionals has been shown to play an important role in patient safety [30]. There appears to be a communication problem regarding information transfer from the doctor through the nurse to the AN and vice versa. It is the nurse who has to decide what from the AN’s report that is to be taken up on a ward round. With teamwork and a joint ward round, improvement could be possible with direct communication and increased understanding of different professional backgrounds. This suggestion is supported by a study that showed that ANs feel less valued when reporting to physicians compared to nurses and suggest interprofessional team training in NHs [31].

A way of interpreting how ANs perceive their role is through role theory, which provides a framework for understanding how individuals acts and takes roles, based on expectations from society and themselves [32]. In our study, the ANs own expectations lied in taking the role of the observer whilst their position entailed a delimited responsibility in contrast to primarily the nurse. In observing, the appropriate action when identifying side effects was according to their social role typically to report it to the nurse. In addition, their social role did not imply participation in ward rounds, a task assigned to the nurse, and even though we found a generally positive attitude towards attending, no signs of attempts to violate their social role existed. According to role theory, conforming to the social role is satisfactory and has the anticipation of a reward whilst violation could lead to sanctions [32]. Hence, for ANs to participate in ward rounds, it needs to be defined as part of their chores as it would then be part of their social role to participate.

### Perspectives on pharmacotherapy

Although good self-perceived knowledge of medications was reported in both the survey and the interview, different indications of lacking knowledge appear among the interview respondents. Examples of this were the notion that paracetamol is dangerous, which is an incorrect assumption [33], or the unfamiliarity with PIMs in older people. This was also seen in the survey where roughly only half of the respondents expressed good self-perceived knowledge on side effects and PIMs. Similarly, a recent study found good self-perceived clinical competence although a much lower level of actual clinical competence [34]. An investigation on behalf of the Swedish Ministry of Health and Social Affairs from 2019 showed an overall lack of medical knowledge among ANs [16]. Moreover, a report from the Swedish Health and Social Care Inspectorate from 2023 noted several deficiencies regarding the health care of older people [35], among these was a considerable prescription of PIMs that should be avoided in older people due to them being extra prone to side effects [36].

An attempt to bring down the treatment with PIMs could include the contribution from ANs with nursing measures instead of medications. However, one study on the beliefs and attitudes towards polypharmacy and medication use among NH staff showed that healthcare staff were more positive to pharmacological management of behaviours of concern such as physical aggression than nurses were [37]. Hence, nursing measures may be more motivating for the ANs if they are informed by the doctor about the risks of DRPs. This suggestion is supported by a qualitative study on perspectives on deprescribing in NHs that found factors such as trust among team members, interprofessional education and interprofessional teams to be important for achieving a successful reduction in dosage or number of medications [38].

### Approaches to knowledge

We found that less than half of the survey respondents had received in-service training on subjects related to medications, side effects and older people and medication sensitivity. There were only a few additional comments elaborating on their thoughts. In the interviews, additional reflections regarding in-service training were obtained although a lower degree of reflection than expected existed on the need for relevant education to adjust for shortcomings detailed in the background, possibly due to limited knowledge [39]. However, respondents of both the survey and interviews were positive towards attending doctor’s ward rounds. Likewise, previous literature supports a positive attitude among ANs towards interdisciplinary practice [40].

Several studies from southern Sweden have shown that medication reviews in teams consisting of a pharmacist, doctor, nurse and, in some cases, ANs and CAs at NHs, can be a way to reduce the number of residents with PIMs and thereby improve the quality of medication treatment in older people and can also reduce hospital readmissions [27, 41, 42]. This supports our suggestion of involving the AN in the doctor’s ward rounds as an opportunity for learning about medications and DRPs. One review of how interventions such as medication review, multi-disciplinary team meetings, staff education and computerised clinical decision could optimise medication treatment in NHs showed that medication appropriateness could be improved but that no effect was seen regarding clinical outcomes [43]. This perhaps reflects the need for continuous learning rather than interventions to achieve an actual clinical impact, which would support a working method that promotes in-service training. Also, this is a feasible approach for increasing knowledge considering the context with understaffing.

**Fig. 5:**
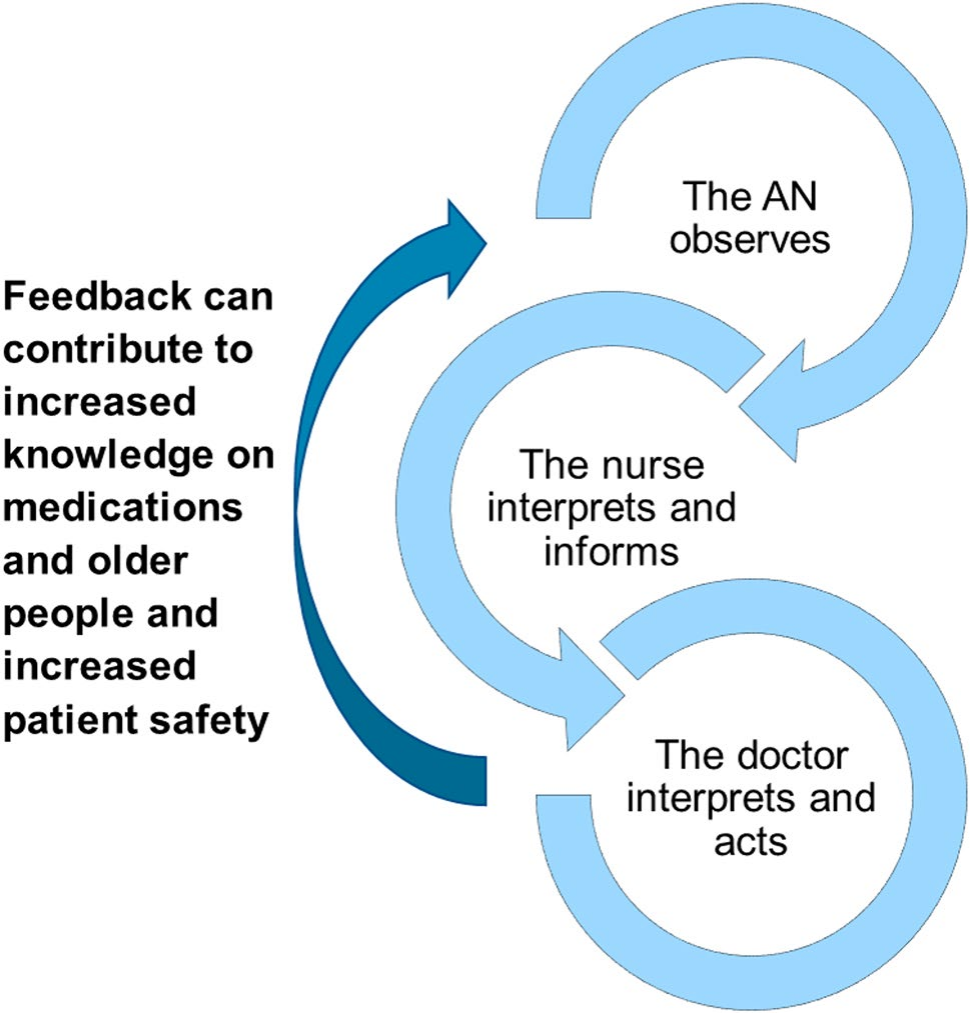
Working method with interprofessional collaboration. Illustration of how a working method with interprofessional collaboration can increase knowledge among ANs in NHs

We suggest a working method where ANs are continuously involved in ward rounds work (Fig. 5). Furthermore, where the importance of the AN for providing accurate information about the well-being of the residents is acknowledged and they are informed about planned follow-up after medication changes and what needs to be observed. The ward rounds should be used as occasions for continuous education where the doctor can explain to the ANs why certain symptoms lead to changes in the medication, which medicines are risky and why and explain the risk-benefit assessments involved in the decision-making. Non-pharmacological nursing measures can be encouraged. Also, we suggest that the AN should participate in multiprofessional medication reviews for residents they work especially close to, and beforehand systematically assess the occurrence of any side effects of medications together with the nurse.

### Strengths and weaknesses

One of the study’s strengths was provided by its mixed methods approach, which gave us the opportunity to deepen the survey results with a different methodology and confirm some of the survey results as well as further explore the study’s aim. The survey dropout was small and data collection took place in several different municipalities of varying size, which gave a broad representation. Triangulation was carried out by having the interviews read by several authors and consensus on meaningful units and coding could thus be reached. A joint analysis was then carried out, which further ensured that the results were interpreted uniformly, in order to achieve trustworthiness [24]. The pre-understanding of the authors is found in the experience of working clinically at NHs and in the research area of medication safety. Furthermore, the authors’ pre-understanding is broadened by including both physicians and a nurse.

Nonetheless, weaknesses of the study are also present. The survey was not tested for reliability but for feasibility in a pilot study. There were some conflicting responses which may be due to the survey not being validated. It could be argued that more questions should be of mixed positive and negative endpoints to lower the risk of response set or answering questions in the same way regardless of its content. This may have reduced the risk of loitering-related response bias. However, some studies argue that it in fact can result in less accurate responses [44]. It could also have been beneficial to have a more neutral response option in the middle of the Likert scale than partially agree, which can be considered leaning in the positive direction. Although few participants stated that they did not speak or understand Swedish fluently, there is still the possibility that some participants had difficulty understanding and interpreting the survey questions, which were sometimes long and complex. In the interview part, there was a risk of selection bias when the NH managers selected the respondents. Furthermore, even though a mixed method was applied, the actual number of participants was limited, and recruitment was confined to southern Sweden. Even though the NHs are representative for a Swedish context regarding laws and policies in the area, the degree of transferability must always be considered by the reader.

### Clinical implications

Better collaboration and feedback contribute to knowledge and reflection that can make the ANs better at observing symptoms of DRPs, which can contribute to patient safety. We propose a working method where the AN is included as a natural part of the ward rounds collaboration and medication reviews. Cooperation and feedback within the framework of doctor’s ward rounds at NHs is an excellent opportunity to increase the knowledge of ANs and utilise their knowledge of patients’ well-being for safe medication follow-up. Seizing this opportunity for learning should be both feasible to implement and cost-effective. The impact on clinical outcomes of the measures needs to be examined in further research.

## Conclusions

The AN’s perceived their professional role in preventing DRPs in NH as being the nurse’s eyes and ears for patient safety. Generally, the ANs’ self-perceived knowledge of medicines was good, although self-perceived knowledge regarding DRPs, and the physiology of older people, was sparser. A positive attitude towards attending rounds to contribute firsthand information on the residents, as well as the opportunity to receive knowledge, existed among the respondents. Based on the findings we propose a working model with interprofessional collaboration, to increase the knowledge of ANs in NHs as well as patient safety.

## Electronic supplementary material

Below is the link to the electronic supplementary material.

Supplementary Material 1

Supplementary Material 2

## Data Availability

The datasets generated and analysed during the current study are not available due to large text files based on individual interviews or surveys but are available from the corresponding author upon reasonable request.

## Declarations

### Authors’ information

All authors have experience from working in nursing homes. ST, BBB, GC, AM, PM and SM are all primary care physicians. ÅC is a registered nurse. BBB, GC, ÅC, PM and SM all lecture on care for older people. SM has extensive experience in medication safety for older people and has regional responsibility for this area in Region Skåne.

## Abbreviations

AN: Assistant nurse
CA: Care assistant
DRP: Drug-related problem
NH: Nursing home
PIM: Potentially inappropriate medicine
