# Supplementary material for: Being the nurse’s eyes and ears: a mixed methods study of assistant nurses’ perceptions of their role regarding drug-related problems in nursing homes
Source: BMC Nurs. 2025 Jun 27;24:670. doi: 10.1186/s12912-025-03416-y (PMC12203716; doi:10.1186/s12912-025-03416-y)
Supplement: Supplementary file 1 — Supplementary Material 1 [file 12912_2025_3416_MOESM1_ESM.docx]

**Appendix 1**

**Survey questionnaire**

**Background information**

Here we ask you to answer questions about you and your background.

1. What is your legal sex?

- Female
- Male

2. How old are you?

- Younger than 25
- 25 – 34
- 35 – 49
- Older than 50

3. Do you have education as an assistant nurse?

- Yes
- No

4. How many years of experience do you have in your occupation?

- Less than 1 year
- 1 – 5 years
- 6 – 15 years
- More than 15 years

5. Do you have delegation for distributing medications?

- Yes
- No

6. Do you have additional training (other than training available at the workplace) in elderly?

- Yes
- No

7. Do you work at a dementia department or at a regular department?

- Dementia department
- Regular department

8. Do you speak and understand the Swedish language unimpeded?

- Yes
- No

9. Do you have permanent or temporary employment?

- Permanent employment
- Temporary employment

**Questions about knowledge**

Here we ask you to answer questions about how you yourself experience your own knowledge about the elderly, medicines, and medication side effects.

|  | *Tick the box that best matches your opinion.* | 1 (Strongly disagree) | 2  (Disagree) | 3  (Partially agree) | 4  (Agree) | 5 (Strongly agree) | Not relevant/Do not wish to respond |
| --- | --- | --- | --- | --- | --- | --- | --- |
| 1. | I feel confident with my knowledge of the medications that my resident takes. |  |  |  |  |  |  |
| 2. | I feel confident with my knowledge of side effects that may occur because of medications. |  |  |  |  |  |  |
| 3. | I often feel worried that problems will arise when new medicines are introduced. |  |  |  |  |  |  |
| 4. | I believe it is important to know which medicines my resident is taking and why. |  |  |  |  |  |  |
| 5. | I feel confident with my knowledge of which medication groups are classified as potentially inappropriate for the elderly |  |  |  |  |  |  |
| 6. | I feel confident with my knowledge of which medication groups pose a risk of causing falls. |  |  |  |  |  |  |
| 7. | I feel confident with my knowledge of which medicines that can pose a risk and need to be paused (after doctor's consultation) in case of stomach upset or high fever, i.e., in case of risk of dehydration/acute kidney failure. |  |  |  |  |  |  |
| 8. | I feel confident with my knowledge of the pros and cons of sleeping medication. |  |  |  |  |  |  |
| 9. | I feel confident with my knowledge of other methods (apart from medications) that can be used to improve sleep in the elderly. |  |  |  |  |  |  |
| 10. | I feel confident with my knowledge of other methods (apart from medication) that can be used to reduce anxiety in the elderly. |  |  |  |  |  |  |
| 11. | I have knowledge of how malnutrition can be detected and prevented in the elderly. |  |  |  |  |  |  |
| 12. | I believe that medication is the most important treatment available for symptoms that can occur with ageing. |  |  |  |  |  |  |

13. Have you ever suspected a medication side effect in the residents you work with?

- Yes
- No

14. Have you ever reported a suspicion of a medication side effect in residents (e.g. to the nurse)?

- Yes
- No

15. Do you participate in multiprofessional drug reviews? (Structured evaluation of a residents’ treatment where a pharmacist participates before/during the meeting)

- Always
- Often
- Sometimes
- Seldom
- Never

|  | *Tick the box that best matches your opinion.* | 1 (Strongly disagree) | 2  (Disagree) | 3  (Partially agree) | 4  (Agree) | 5 (Strongly agree) | Not relevant/Do not wish to respond |
| --- | --- | --- | --- | --- | --- | --- | --- |
| 16. | I believe it is important to participate in medication reviews to share my experiences of the resident’s well-being. |  |  |  |  |  |  |
| 17. | I believe it is important to participate in medication reviews in order to take part in the doctor's/pharmacist's stance on the resident. |  |  |  |  |  |  |
| 18. | I believe that I learn something from participating in medication reviews. |  |  |  |  |  |  |

19. Do you participate in ward rounds visits with doctors?

- Always
- Often
- Sometimes
- Seldom
- Never

20. If seldom/never – why not?

- Lack of time
- Not invited
- Do not have anything to contribute with
- Other, namely:

____________________________________________________________________________________________

|  | *Tick the box that best matches your opinion.* | 1 (Strongly disagree) | 2  (Disagree) | 3  (Partially agree) | 4  (Agree) | 5 (Strongly agree) | Not relevant/Do not wish to respond |
| --- | --- | --- | --- | --- | --- | --- | --- |
| 21. | I believe it is important to participate in ward rounds visits to share my experiences of the resident’s well-being. |  |  |  |  |  |  |
| 22. | I believe it is important to participate in ward rounds visits in order to take part in the doctor's stance on the resident. |  |  |  |  |  |  |
| 23. | I believe that I learn something from participating in ward rounds visits. |  |  |  |  |  |  |

**Questions about in-service training**

Here we ask you to answer questions about what in-service training (further education) you have received at your workplace, and what in-service training you wish in the future.

1. Have you been offered introductory training at the workplace?

- Yes
- No

2. Did you receive training in connection with receiving delegation for distribution of medications?

- Yes
- No
- Do not have delegation

3. Have you attended in-service training in the last year?

- Yes
- No

4. If no – why not?

- Have not been offered
- Have not had time
- Have not been allowed to attend during work hours
- Have already knowledge on the theme for the in-service training
- Other, namely:

_____________________________________________________________________

5. Have you been offered in-service training on medication management?

- Yes
- No

6. If yes – in what form?

- In-person lecture
- Digital lecture
- Online education
- Other, namely:

_____________________________________________________________________

7. Have you been offered in-service training about medication side effects?

- Yes
- No

8. If yes – in what form?

- In-person lecture
- Digital lecture
- Online-education
- Other, namely:

_____________________________________________________________________

9. Have you been offered in-service training about age-related changes in the organs that affect medication sensitivity?

- Yes
- No

10. If yes – in what form?

- In-person lecture
- Digital lecture
- Online-education
- Other, namely:

_____________________________________________________________________

11. Have you been offered other in-service training related to ageing (e.g. dealing with dementia or similar)?

- Yes
- No

12. Do you want in-service training to a different extent than today?

- More
- Less
- Unchanged

13. How do you want in-service training? Multiple answer options possible.

- In-person course
- Online course
- Daily discussions with authorized personnel
- Following a nurse
- Other, namely:

_____________________________________________________________________

14. Do you have further thoughts about in-service training that you would like to share with us:

______________________________________________________________________________________

______________________________________________________________________________________
