# Supplementary material for: Being the nurse’s eyes and ears: a mixed methods study of assistant nurses’ perceptions of their role regarding drug-related problems in nursing homes
Source: BMC Nurs. 2025 Jun 27;24:670. doi: 10.1186/s12912-025-03416-y (PMC12203716; doi:10.1186/s12912-025-03416-y)
Supplement: Supplementary file 2 — Supplementary Material 2 [file 12912_2025_3416_MOESM2_ESM.docx]

**Appendix 2**

**Semi-structured interview guide**

**Overarching themes**

Tell us about how you feel that your knowledge of medication treatment in the elderly is in relation to the tasks and responsibilities you have for your residents.

Tell us about how you got your medication distribution delegation?

**Checklist (=has this been addressed?)**

1. Different types of potentially harmful medications
2. To identify and report side effects
3. Ageing and medication sensitivity
4. In-service training
5. Wishes regarding future in-service training, subjects and form
6. Participation in ward rounds and medication reviews
7. View of own part in patient safety

**Termination**

1. What do you think is the most important of what was said?
2. Summary
3. Have we missed something/point of view that has not been touched upon?
